# Mental health service utilization among people with intellectual and developmental disabilities and serious mental illness before and during the emergence of telehealth services

**DOI:** 10.3389/frhs.2026.1832994

**Published:** 2026-06-01

**Authors:** Emily Lauer, Renata E. Howland, Julie Royer, Jean P. Hall, Noelle K. Kurth, Suzanne L. Hunt, Dawn Walter, Charles J. Neighbors, Suzanne W. McDermott

**Affiliations:** 1 Department of Family Medicine & Community Health, UMass Chan Medical School, Worcester, MA, United States; 2 Eunice Kennedy Shriver Center, UMass Chan Medical School, Worcester, MA, United States; 3 Department of Population Health, NYU Grossman School of Medicine, New York, NY, United States; 4 SC Revenue and Fiscal Affairs Office, Data Integration and Analysis Division, Columbia, SC, United States; 5 Institute for Health and Disability Policy Studies, University of Kansas, Lawrence, KS, United States; 6 Department of Biostatistics and Data Science, University of Kansas Medical Center, Kansas City, KS, United States; 7 Department of Psychiatry, NYU Grossman School of Medicine, New York, NY, United States; 8 Department of Environmental, Occupational, and Geospatial Health Science (EOHS), City University of New York, Graduate School of Public Health and Health Policy (CUNY-SPH), New York, NY, United States

**Keywords:** intellectual disability, medicaid, mental health services, mental illness, telehealth

## Abstract

**Introduction:**

Few populations face more disadvantage than those with lifelong intellectual and developmental disabilities (IDD) and those with serious mental illness (SMI). People with IDD may face unique challenges in the manifestation and treatment of SMI; little is known about these challenges during the widespread expansion of telehealth mental health services during the COVID-19 pandemic which disrupted service availability.

**Methods:**

Using Medicaid claims from Kansas, Massachusetts, New York and South Carolina, mental health service utilization patterns for three cohorts of people ages 1–45 years were studied: those with IDD, those with SMI, and those with SMI and IDD. Utilization was examined before (2018–2019) and during (2020–2021) the emergence of telehealth services for each cohort. Meta-analysis was used to compare odds of mental health service utilization by demographic subgroups.

**Results:**

The prevalence of mental health service utilization was approximately 75% for the IDD/SMI cohort, 60% for the SMI cohort, and 30% of the IDD cohort in 2018. Teens 13–17 years and young adults tended to have the highest levels of service utilization. Service utilization was driven by different diagnoses for the groups. The SMI cohort utilized services significantly more for mood and anxiety disorders, and the IDD cohort utilized services significantly more for comorbid neurodevelopmental conditions, anxiety, and trauma-related disorders. The IDD/SMI cohort utilized services more bipolar and related disorders and had a younger median age of service utilizers for trauma- and stress-related disorders than the SMI cohort.

**Discussion:**

The IDD/SMI cohort had the highest mental health service utilization rates compared to the other two cohorts, with minimal urban-rural differences, suggesting mental health services may be reaching those at the highest levels of risk for adverse outcomes. People with IDD demonstrated substantially lower rates of telehealth utilization for mental health needs; however, people in the cohort with IDD and SMI demonstrated similar or higher rates (in adults) of telehealth utilization compared to people with SMI only. Even with expanded telehealth services, the COVID-19 pandemic appeared to partially disrupt utilization across all cohorts and age groups. Findings suggest that people with IDD and SMI experience trauma- and stressor-related disorders that require treatment at younger ages than people with SMI only.

## Introduction

The public health and health services perspective on at-risk populations has expanded in recent years to focus on people who experience intersectionality of multiple risks. Few populations face more disadvantage than those with lifelong intellectual and developmental disabilities (IDD) and those with serious mental illness (SMI). Both groups are disproportionately poor, represent diverse ethnic and racial minorities, live in unstable housing, lack consistent health care, and experience substantial deficiencies in their social and health services (1–4). This study examined the utilization of mental health services for people with IDD, with SMI and with both IDD and SMI before and during the emergence of telehealth services during the COVID-19 pandemic, a time during which mental health services were disrupted, and service access challenges were potentially magnified.

People with IDD may face unique challenges in the manifestation and treatment of serious mental illness (SMI), including stress from life changes, communication difficulties, and limited professional training for mental health professionals. However, there are limited studies on mental health service utilization among those with IDD and SMI. Moreover, few studies have investigated how patterns of service access may have changed during the COVID-19 pandemic and the associated expansion of telehealth services, which may be a complicated modality for people with IDD. This information is crucial for understanding and improving the treatment and management of mental health conditions in this population, aligning with initiatives like Healthy People 2030. Medicaid data is a promising way to address these gaps because most individuals with IDD and SMI are Medicaid eligible, providing a comprehensive data source on their service utilization.

The prevalence of intellectual disability (ID) in the United States is estimated to be around 1%–2%, based on surveillance programs administered by the National Center for Health Statistics (NCHS) and the Centers for Disease Control and Prevention (CDC). The prevalence varies depending on the specific criteria used to define intellectual disability and the sources of data. In addition, the CDC estimates the 2021 prevalence of autism represents 2.4% of the population (1 in 44 children) (5). Among children with autism, it is estimated that around one-third have an ID (defined as an IQ score of 70 or below), thus the prevalence of children in the U.S. with both autism and ID is roughly 1 in 132 (0.76%). The combined prevalence of ID and autism is often referred to as IDD, which includes some additional conditions such as genetic conditions like Down Syndrome, cerebral palsy, neural tube defects, and some brain injuries and infections during childhood. The onset of IDD is usually prenatal although many of the conditions do not manifest until early childhood (6, 7).

The CDC estimates 1 in 20 US adults lives with a serious mental illness (SMI), such as schizophrenia, bipolar disorder, or major depression (4). SMI usually manifests during adolescence and young adulthood, although research shows that parents report 16.5% of youth/adolescents experience a mental health condition (8). Additionally, there are numerous reports of the co-occurrence of IDD and SMI including major depression, bipolar disorder, psychotic disorders, and impulse control disorders (9), with some studies suggesting that psychiatric disorders may be three to four times more prevalent in people with IDD compared to the general population (10). Studies using the National Core Indicators-In Person Survey (NCI-IPS), which uses representative samples of people with IDD who use state-funded services in the U.S., found rates of IDD with co-occurring mental health conditions ranging from 37% in 2009–2010 (11) to 45% in 2012–2013 (12).

SMI may manifest differently in people with IDD, compared with the general population. The National Association for the Dually Diagnosed, in collaboration with the American Psychiatric Association, compiled the Diagnostic Manual–Intellectual Disability, to describe how psychiatric diagnosis can be adapted in working with people with IDD. When the DSM-5 was put into widespread use, NADD developed DM-ID-2 specifically about people with IDD and mental illness (13). For children and adults with IDD, the effects of changing living arrangements, high turnover in staff, limited contact with family, seeing siblings and others achieve adult milestones, and lack of structure when public school ends at age 21 can be highly stressful events, which can precipitate or heighten mental illness signs and symptoms. Additionally, treatment of SMI in people with co-occurring IDD can be complex and training for mental health professionals is sparse regarding the needs of this population (14). Receptive and expressive communication challenges, as well as challenges with executive functions may affect the success of non-pharmaceutical mental health treatment modalities for people with IDD.

Most individuals with IDD with co-occurring serious mental illness (SMI) are Medicaid eligible, either through Home and Community-based Service (HCBS) waivers or standard Medicaid. The HCBS Medicaid waiver provides long-term services and supports in the community to persons with IDD who would otherwise require an institutional level of care in an intermediate care facility for IDD (ICF-DD). Most states finance HCBS services through the Section 1915 (c, i, k) Medicaid waiver programs which cover community-based habilitative interventions and residential support programs but not all these state programs cover psychiatric care benefits. In many cases psychiatric benefits must be accessed through the individual's broader eligibility for Medicaid benefits (state plan Medicaid), with use of medical necessity criteria to determine the level of care provided.

In most states, the state developmental disability agency is separate from the state mental health agency. Psychiatric hospital inpatient units and community residential and day programs typically have limited acute psychiatric services that are designed to serve people with psychiatric conditions that co-occur with autism or IDD. The intersectionality of comorbid IDD and SMI with poverty, unstable housing, minority status, and challenges with medical and social supports, makes Medicaid an important data source to understand the utilization of mental illness services for people with IDD, SMI, and those with both IDD and SMI (15, 16).

Treatment and ongoing management of serious mental illness is critical for the maintenance of overall health. People with mental health conditions are at higher risk for chronic medical conditions, and serious mental illness increases the risk, occurrence, progression and adverse outcomes associated with these chronic health conditions (17). Conversely, positive mental health, which is possible for most people with serious mental illness, is associated with physical health improvements*.* These connections are part of the reason the federal Healthy People 2030 initiative includes a focus on treatment for mental health conditions, including adults with serious mental illness, and ensuring children and adolescents with mental health problems get treatment (18).

**Figure 1:**
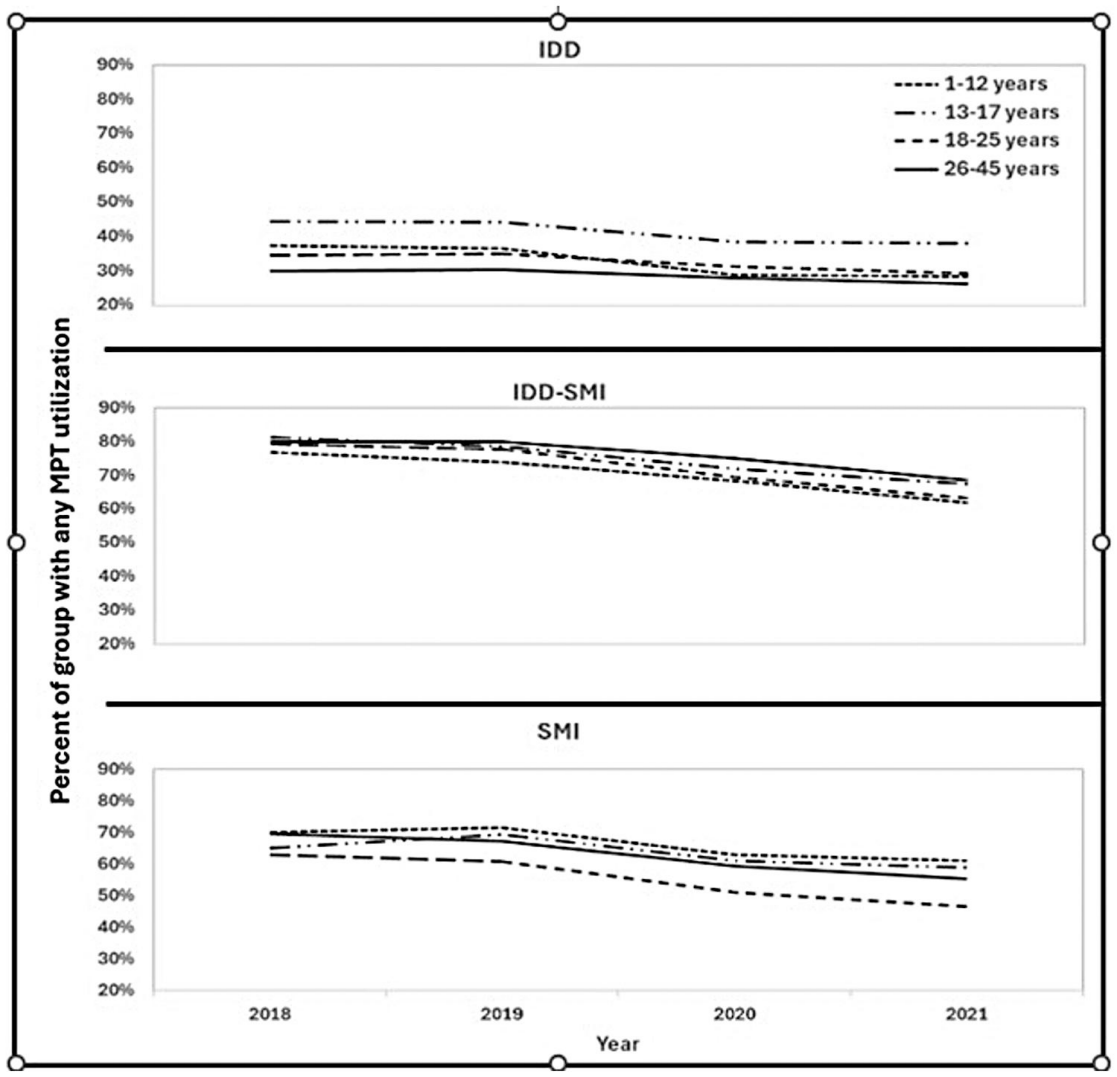
Percent of cohort members with any mental health service utilization, by cohort, age group and year

**Figure 2:**
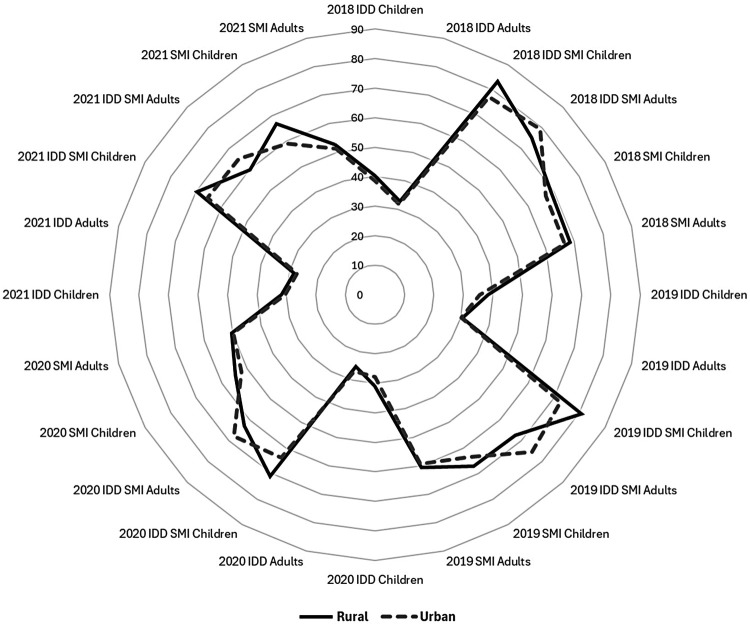
Percentage of members with Any mental health utilization by cohort, year, urban vs. rural, and adults vs. children. Includes all Places of Service (Inpatient, Intensive Outpatient, ED, Outpatient and Telemedicine).

There is a gap in the existing literature related to utilization of psychiatric services for people with IDD and those with both IDD and SMI, compared to people with SMI only. This study aimed to address this gap by analyzing the utilization of mental health services among a cohort of children and adults insured by Medicaid, from childhood through age 45 years, who have IDD, SMI, and combined IDD and SMI both before and during the emergence of telehealth services during the COVID-19 pandemic. We studied service utilization data from four states, with different Medicaid state plans and diverse population characteristics, to capture the variability in the members who did not have another insurance plan. We hypothesized that people with IDD/SMI would have lower mental health service utilization than people with SMI, but higher utilization compared to people who only had IDD (related to Figure 1). The study design allowed us to test this hypothesis both in a period before the emergence of telehealth services, 2018–2019, and during the first two years of telehealth's substantial emergence, 2020–2021. In this latter period, states shifted to permit telehealth outpatient services for mental health (19) during the COVID-19 pandemic. Our secondary hypothesis included that mental health service utilization is higher among all cohorts in urban areas compared to rural areas because of limited provider access (20, 21), but that this effect may be mitigated by the expansion of telehealth services (related to Figure 2). We hypothesized that telehealth utilization would be lower in the IDD and IDD and SMI cohorts compared to people with SMI, related to differences in cognitive and communication abilities (related to Table 2). Lastly, we hypothesized that children with SMI and children with IDD and SMI would have more similar prevalence of mental health service utilization than adults aged up to 45 years (related to Table 2).

## Materials and methods

### Study population

To identify people for each of three cohorts—people with IDD, people with SMI, and people with both SMI and IDD—Medicaid fee-for-service and managed care encounter claims from Kansas, Massachusetts, New York and South Carolina were searched for IDD and SMI diagnosis codes, who were 1–45 years old during a case-finding period from 2015 to 2019, selecting only people with at least 11 months of continuous eligibility. People who were dually eligible for Medicaid and Medicare, or who had Medicaid and other private insurance were excluded because Medicaid claims would provide an incomplete measure of service utilization. Similar to prior work (22), codes used to define IDD were selected from the following potentially disabling condition algorithms from the Centers for Medicare and Medicaid Services Chronic Conditions Data Warehouse including: 1) intellectual disabilities and related conditions, 2) autism spectrum disorder and 3) cerebral palsy (23). Codes used to define SMI were selected from the following Agency for Healthcare Research and Quality Clinical Classification groupings: 1) schizophrenia spectrum and other psychotic disorders, 2) bipolar and related disorders and 3) depressive disorders (24). Members were included in one of the three cohorts (IDD only, SMI only, or both) if they had one inpatient stay or two outpatient non-pharmacy/non-laboratory services with respective diagnoses separated by at least one month.

This work was regarded either as exempt by Institutional Review Boards, or that review by an IRB was not required in some states because they were using de-identified data for the purposes of health surveillance.

### Primary outcome

Mental health utilization (MPT) was measured in a subsequent period (calendar years of 2018, 2019, 2020 and 2021) for the three cohorts for members with at least 11 months of eligibility in each year. Mental health utilization followed the 2021 Healthcare Effectiveness Data and Information Set (HEDIS®) specifications (HEDIS® is a registered trademark of the National Committee for Quality Assurance (NCQA) (25). The MPT measure provided a standardized method for comparing utilization across states and over time. We examined any mental health service utilization and utilization by place of service: inpatient, intensive outpatient or partial hospitalization, outpatient, Emergency Department (ED), or telehealth. The full specifications are available from HEDIS®. Broadly speaking, we identified all claims with a mental health diagnosis code. Then, we used procedure codes, revenue codes and place of service to define the service type. We revised this measure for purposes of our study by excluding autism spectrum disorders from the list of mental health diagnoses and queried primary and secondary diagnoses up to five fields. The percentage of members receiving services was calculated for each state and then averaged.

### Demographic variables

Additional demographic information on individuals was pulled from each state's Medicaid eligibility files, including age at first enrollment, sex (male/female), and number of months of enrollment over the study period. We limited our adult age group to a cut-off of 45 years, since there is a substantial decline in Medicaid-only members after this age. Given the interest in rural/urban differences, we used member zip code at cohort entry and linked that zip code with Centers for Medicare and Medicaid Ambulance Fee Schedule Public Use Files (27). An individual who was living in a rural or super rural zip code was coded as rural, and those without a designation were coded as urban. If member zip was missing rural/urban field was set to unknown.

### Statistical analyses

MPT utilization was subcategorized by the type of utilization: inpatient services, intensive outpatient services, Emergency Department, outpatient, and telehealth. Telehealth visits were identified by using one of the following: 1) current procedural terminology modifier “95” or “GT” or 2) telehealth provided other than in patient's home or place of service “02”. Place of service “10” or telehealth provided in patient's home effective date was January 1, 2022 and therefore was not used in current analysis (28). Data across the states was combined (averaged) but was not weighted for population size to ensure one state's patterns didn’t outweigh other states. State-specific patterns were examined prior to combination to identify any extremely divergent patterns. Pre-COVID time periods were defined as utilization in calendar years 2018–2019. COVID-era utilization was defined as calendar year 2020–2021. Underneath the MPT measure definition, the individual SMI-related ICD-10 diagnoses relating to each instance of utilization were also examined in each state and ranked. The Clinical Classifications Software Refined (CCSR), sponsored by the Agency for Healthcare Research and Quality, was used to group the diagnoses into clinically meaningful categories (29).

Logistic regressions were conducted to assess the association of mental health service utilization and demographic factors. Each state modeled the use of mental health services using logistic regression to assess associations with age, gender and rural/urban location. Each state conducted their own regression model using common methods and variable structure, one for 2018 and one for 2021. We used a second-stage approach to combine the results of the regressions and standardize the effect sizes by computing the log odds ratios and estimating upper and lower bounds (30). We conducted meta-analyses using R's “metagen” package (31). We applied a random-effects model to allow for variation in the underlying populations between states (32). To determine if any of the data points distorted the final model, we used the leave-one-out method, wherein the results are recalculated following the exclusion of each state (31).

Analysts from each state compiled data from their individual Medicaid databases. Importantly, a data management plan and the same statistical code was shared across the state teams to ensure the methods of analysis were consistently applied. Aggregate data from each site was captured in pre-formatted data tables and results shared among states and viewed alongside pooled results. One central analyst combined the results across states. We followed a version of the HEDIS measure for assessing mental health utilization that was modified in the same way for all states; it is possible that individual state differences in claim coding procedures may have had small effects on cross-state consistency as Medicaid claims data are intended to facilitate payment and not measure the health of plan members. Analyses, except for the meta-analysis, were conducted with SAS 9.4 (26). One figure was also created in R.

## Results

Table 1 shows the characteristics of the 636,166 Medicaid members, ages 1–45 years in the three cohorts, from four states. Most people (71%) were in the SMI only cohort; 23% were in the IDD only cohort, and 5% were in both categories. The IDD only cohort was much younger than the other cohorts, with 48% between the ages of 1 and 12 compared to 6.5% in the cohort with only SMI. People in the IDD cohort were also much more likely to be male (69% vs. 31%). The cohort of people with both IDD and SMI was more like the IDD cohort in terms of sex (with 60% male) but more like the cohort with only SMI in terms of age distribution. In all three cohorts, the majority (>87%) of the population lived in urban areas.

**Table 1:** Characteristics of medicaid-insured individuals ages 1-45 years with IDD, SMI or both conditions in Kansas, New York, South Carolina and Massachusetts from 2018.

Characteristic	IDD only		IDD/SMI		SMI only		Total	
	*N*	%	*N*	%	*N*	%	*N*	%
Total	148,961	100%	33,229	100%	453,976	100%	636,166	100%
Sex								
Female	46,369	31.1%	13,183	39.7%	294,397	64.8%	353,949	55.6%
Male	102,592	68.9%	20,046	60.3%	159,579	35.2%	282,217	44.4%
Age								
1–12 years	71,043	47.7%	2,168	6.5%	15,857	3.5%	89,068	14.0%
13–17 years	23,434	15.7%	5,118	15.4%	51,599	11.4%	80,151	12.6%
18–25 years	23,637	15.9%	8,909	26.8%	84,904	18.7%	117,450	18.5%
26–45 years	30,847	20.7%	17,034	51.3%	301,616	66.4%	349,497	54.9%
Rural/Urban								
Rural	16,301	10.9%	3,580	10.8%	51,861	11.4%	71,742	11.3%
Urban	132,061	88.7%	29,518	88.8%	397,782	87.6%	559,361	87.9%
Missing/Unknown	599	0.4%	131	0.4%	4,333	1.0%	5,063	0.8%

IDD, intellectual and developmental disabilities; SMI, serious mental illness.

Individuals with IDD and SMI were identified using a combination of procedure and diagnoses codes based on established classifications.

Age was estimated as of January 1, 2018.

Rural/urban was defined based on residential zip code.

We examined the differences in mean and median ages in each cohort by state to understand differences between the cohorts and consistency across states (data not shown). The cohort-specific patterns across the states were consistent in that the IDD cohort was the youngest on average (medians ranged between 11 and 14 years) skewed toward younger ages. The IDD SMI cohort was the next oldest group (medians ranged between 21 and 31 years), also skewed toward younger ages. The SMI cohort was the oldest across states (medians ranged between 18 and 38 years) and not skewed in either direction.

The prevalence of any mental health service utilization for the IDD/SMI cohort was approximately 75% for all age groups in 2018 (Figure 1). Within this cohort, utilization was largely similar across age groups, ranging from a low of 73.5% among the 1- to 12-year-olds to 76.5% for 13- to 17-year-olds. The proportion of people from the other two cohorts with mental health service utilization was significantly lower (*χ*^2^, *p* < 0.0001) than the IDD/SMI cohort in all states. The SMI only cohort had the next highest utilization at approximately 60% of the cohort in 2018. Lastly, roughly 30% of the IDD only cohort had any mental health service utilization. Within this cohort, individuals ages 13 to 17 had the highest rate of mental health service utilization (39.8% in 2018), whereas individuals 26 to 45 years had the lowest prevalence of utilization (26.9% in 2018).

Across all three cohorts, the rate of mental health service utilization declined over time from 2018 to 2021, both overall and for all settings except telehealth for all cohorts, and for Emergency Department utilization for adults with IDD. Decreases in mental health service utilization were observed for inpatient, intensive outpatient, Emergency Department and outpatient services across all age groups and cohorts during the COVID-19 pandemic (Table 2) except for adults with IDD. The largest decreases in mental health service utilization were in outpatient service use for 18–45-year-olds with SMI (33% drop in utilization) and the 18–45-year-olds with IDD/SMI (26.5% drop in utilization). These changes mostly appeared to be offset by increases in telehealth utilization. In most states, people with IDD with mental health service utilization were on average younger than the cohort overall. For all cohorts and age groups in 2018 and 2019, outpatient utilization was the most common mental health service setting, followed by the Emergency Department (Table 2). In 2020 and 2021, this pattern shifted such that telehealth became the second most common service type, with the Emergency Department shifting to the third-most common. The use of telehealth appeared to replace some outpatient service utilization in all cohorts, and some Emergency Department utilization in all cohorts except those with IDD only.

**Table 2:** Percent of cohort members with any mental health service utilization, by cohort, age group, type of service and pre-/during the emergence of telehealth.

Cohort	Age group	Service type	2018/2019	2020/2021
IDD ONLY	1–17 years	Inpatient	1.3%	1.1%
		Intensive Outpatient	2.2%	1.2%
		Outpatient	37.8%	28.9%
		Emergency Department	2.8%	2.4%
		Telehealth	0.4%	9.4%
	18–45 years	Inpatient	0.8%	0.9%
		Intensive Outpatient	1.8%	1.2%
		Outpatient	31.2%	25.0%
		Emergency Department	2.8%	2.8%
		Telehealth	0.3%	9.8%
IDD/SMI	1–17 years	Inpatient	15.9%	8.8%
		Intensive Outpatient	10.9%	5.7%
		Outpatient	76.2%	61.7%
		Emergency Department	22.0%	15.5%
		Telehealth	3.5%	35.0%
	18–45 years	Inpatient	11.2%	8.1%
		Intensive Outpatient	12.2%	9.1%
		Outpatient	75.9%	61.2%
		Emergency Department	22.4%	17.3%
		Telehealth	2.5%	33.9%
SMI ONLY	1–17 years	Inpatient	9.7%	5.5%
		Intensive Outpatient	8.8%	6.4%
		Outpatient	67.1%	55.3%
		Emergency Department	15.9%	11.2%
		Telehealth	2.0%	32.8%
	18–45 years	Inpatient	8.4%	5.9%
		Intensive Outpatient	8.8%	5.4%
		Outpatient	62.8%	46.5%
		Emergency Department	15.4%	12.0%
		Telehealth	1.6%	24.3%

IDD, intellectual and developmental disabilities; SMI, serious mental illness.

As shown in Table 3, when we examined the leading diagnoses by cohort over time (pre- vs. post-emergence of telehealth), we found that mental health service utilization for the SMI cohort was largely driven by mood (i.e., depressive) and anxiety disorders, as well as other serious mental illnesses (e.g., bipolar, schizophrenia). For the cohort of people with only IDD, neurodevelopmental disorders [largely attention deficit and hyperactivity disorder (ADHD)] were the leading diagnoses associated with mental health service use among people with such service utilization. While anxiety and notably trauma- related disorders were also commonly associated with service utilization in people with IDD, there were also more diagnoses associated with utilization related to disruptive, impulse-control and conduct disorders for the cohort with IDD compared to the cohort with SMI. The mental health service utilization for people in the IDD/SMI cohort was associated with a combination of diagnoses from the top-ranking clinical categories for each cohort, including both mood (depressive) disorders (as the top ranked conditions), as well as neurodevelopmental disorders. In the cohort with IDD/SMI, the percentage of cohort members using mental health services for bipolar and related disorders was higher (24.1%) than in the SMI cohort (16.7%) (*p* < .0001 in *χ*^2^ analyses for each state and each of the four years). Across cohorts, the top-ranked conditions remained similar over time. However, in the IDD group, service utilization due to mood (depressive) disorders rose in both the percentage of people utilizing mental health services for these conditions, as well as in the relative ranking of top causes of utilization for this cohort in the latter time period which was during the COVID-19 pandemic.

**Table 3:** Top ranked diagnosis groups associated with mental health utilization for each cohort, with percent of members with mental health utilization for each diagnosis.

	2018–2019 average					
	IDD		IDD/SMI		SMI	
RANK	CCSR label	% members	CCSR LABEL	% members	CCSR label	% members
1	Neurodevelopmental disorders	24.5%	Depressive disorders	37.2%	Depressive disorders	47.2%
2	Anxiety and fear-related disorders	7.2%	Anxiety and fear-related disorders	25.5%	Anxiety and fear-related disorders	30.3%
3	Disruptive, impulse-control and conduct disorders	6.4%	Neurodevelopmental disorders	24.1%	Bipolar and related disorders	16.7%
4	Trauma- and stressor-related disorders	4.2%	Bipolar and related disorders	24.1%	Trauma- and stressor-related disorders	15.0%
5	Other specified and unspecified mood disorders	2.4%	Schizophrenia spectrum and other psychotic disorders	23.5%	Schizophrenia spectrum and other psychotic disorders	10.3%

	2020–2021 average					
	IDD		IDD/SMI		SMI	
RANK	CCSR label	% members	CCSR label	% members	CCSR label	% members
1	Neurodevelopmental disorders	18.5%	Depressive disorders	29.1%	Depressive disorders	35.8%
2	Anxiety and fear-related disorders	6.9%	Anxiety and fear-related disorders	22.8%	Anxiety and fear-related disorders	26.9%
3	Disruptive, impulse-control and conduct disorders	4.8%	Schizophrenia spectrum and other psychotic disorders	21.9%	Bipolar and related disorders	14.9%
4	Trauma- and stressor-related disorders	3.8%	Bipolar and related disorders	20.3%	Trauma- and stressor-related disorders	12.9%
5	Depressive disorders	3.2%	Neurodevelopmental disorders	18.1%	Schizophrenia spectrum and other psychotic disorders	10.0%

IDD, intellectual and developmental disabilities; SMI, serious mental illness; CCSR, clinical classification software refined.

To understand the relationship between age and service utilization, we also examined the mean and median age of the people within each cohort utilizing mental health services for various categories of diagnoses (data not shown). Within the cohort with IDD, people using services for neurodevelopmental disorders, miscellaneous mental and behavioral disorders/conditions, and feeding and eating disorders tended to be younger (median age between 7 and 18 years), whereas people using services for Depressive disorders (median ages 17–29 years old), Personality disorders, Obsessive-compulsive and related disorders all tended to be older (median ages 14–30 years old). The cohort of people with IDD and SMI had similar median ages of service utilizers as the SMI cohort. Two areas of moderate differences were observed. The first was for Disruptive, impulse-control and conduct disorders where the median age of service utilizer was younger for people with SMI (14–21 years) compared to people with IDD and SMI (16–31 years). Another condition with younger median age of service utilizers was Trauma- and stressor-related disorders; the mean age of service utilizers in the IDD and SMI cohort ranged from 17 to 29 years, whereas it ranged from 20 to 33 years for people with SMI only.

### Service utilization in urban vs. Rural Settings

As shown in Figure 2, there was generally very little difference in the prevalence of mental health service utilization within any of the cohorts by urban or rural setting across age groups. The exception to this finding was a pattern of small increases in the proportion of children with IDD and SMI who had mental health service utilization in rural areas compared to urban areas across years studied. This utilization pattern was flipped for adults with IDD and SMI, with a slightly greater prevalence of mental health service utilization for adults in urban areas compared to rural areas. In 2021, there was a slightly greater prevalence of mental health service utilization for children with SMI in rural areas compared to urban areas.

### Meta-Analysis

**Figure 3:**
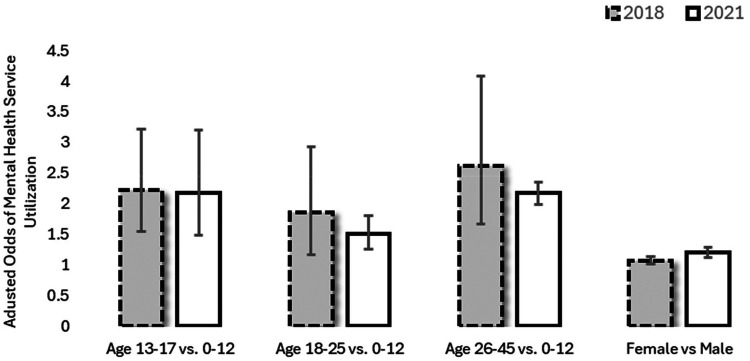
Meta analyses of adjusted odds of mental health service utilization across four states in 2018 and 2021.

In the meta-analysis across states, estimated adjusted odds of mental health service use were calculated controlling for factors of rurality, gender, and age as shown in Figure 3. In random effects models, being female was consistently associated with increased odds of mental health service utilization across the states. This association became increasingly different across genders in 2021 [aOR=1.0737 (1.0198; 1.1305), *p* = 0.0068] compared to 2018 [aOR=1.1989 (1.1201; 1.2832), *p* < 0.0001]. No states were outliers in the meta-analysis, and the difference between genders decreased slightly when controlled for age and rurality. There was no significant difference in mental health service utilization in rural environments compared to urban in either 2018 or 2021, and this was consistent across states. All age groups (13–17 years, 18–25 years and 26–45 years) demonstrated significantly increased (*p* < 0.0001) mental health service utilization compared to people under 13 years old in both 2018 and 2021 across all states. Controlling for gender decreased these odds slightly in all years, though the strong significance of the effects remained. Additionally, the difference in age-related utilization decreased in 2021 compared to 2018. In 2018, there was a greater range of age-related difference across states compared with this youngest age group. However, in 2021, this difference narrowed, with states showing more similar patterns.

## Discussion

This study used Medicaid data from four states during two periods, before and during widespread use of telehealth related to the COVID pandemic, to examine mental health service utilization among those with IDD and SMI. Contrary to our hypothesis, we found that individuals with both IDD and SMI had the highest mental health service utilization compared to either the IDD or SMI only cohorts, with minimal urban-rural differences. These unexpected results suggest that mental health services reached those at the highest levels of risk for adverse outcomes as they were more likely to receive inpatient, Emergency Department, outpatient, and telemedicine services than Medicaid members with SMI or IDD alone.

We hypothesized that telehealth utilization would be lower in the IDD and IDD/SMI cohorts compared to people with SMI related to differences in cognitive and communication abilities. This was partially true, with people with IDD demonstrating substantially lower rates of telehealth mental health utilization; however, people in the cohort with IDD and SMI demonstrated similar or higher rates (in adults) of telehealth utilization compared to people with SMI only, illustrating greater access to this service than expected.

Lastly, we hypothesized that children with SMI and children with IDD and SMI would have more similar proportions of mental health service utilization than adults aged up to 45 years. People with IDD and SMI collectively had a higher prevalence of mental health service utilization across all age groups compared to people with SMI only. However, the COVID-19 pandemic appeared to affect utilization across all cohorts and age groups, both lowering the prevalence of utilization collectively, but also forcing a major shift toward telehealth services with reductions in mental health service utilization prevalence in all other settings.

It is possible that the higher prevalence of mental health service utilization in children with IDD and SMI may be related to the challenges in obtaining SMI diagnoses in rural areas, which have more limited access to clinicians to diagnose these conditions. We theorize that children with more substantial needs are more likely to be diagnosed in urban areas, a geographic reality which, in turn, is associated with a higher prevalence of service utilization.

We observed substantial differences in the conditions driving mental health service utilization among the cohorts. First, ADHD was a leading diagnosis associated with mental health service utilization for the IDD cohort, which is consistent with documented increased prevalence (33) of ADHD among people with IDD. This association is likely driven by the requirements for regular mental health service utilization to access medications used to treat ADHD, which are controlled substances. Additionally, some children and teens who initially presented with IDD in our cohort, appeared to go on in later years to develop serious mental illness, however this was a minority of the cohort.

Secondly, prior research has shown that in people with SMI, the extent of their symptomatology of their mental health conditions tends to drive utilization (e.g., psychological distress, expressive behavior) (34)*.* Our data on the diagnoses associated with service utilization for people with SMI and people with IDD and SMI seem to correspond with these findings.

Thirdly, prior research has suggested that mental health service utilization for people without SMI is largely driven by social factors such as low income and low social supports (34). Our results support these prior findings partially for people with IDD. People with IDD exhibited utilization for some conditions associated with base symptomatology, such as ADHD. However, they also exhibited utilization due to conditions that may be affected or influenced by social factors and environmental factors, such as exposure to non-supportive environments and exposure to abuse; mental health service utilization due to anxiety and fear-related disorders; trauma- and stressor-related disorders; and disruptive, impulse-control and conduct disorders, which may be related to expressive communication needs, environmental stressors, or underlying physical health needs (35, 36)*.* Specifically, findings suggest that people with IDD and SMI experience trauma- and stress-related disorders that require treatment at younger ages than people with SMI only. Other differences in mean age of service utilizers, for conditions such as schizophrenia, age differences among utilizers were expected as these mental health conditions have later onset and would not be present in young children.

We observed an overall decrease in service utilization due to disruptive conditions during the COVID-19 pandemic. This disruption may be related to lower availability of school-based services, particularly in two of the states, although this association could not be directly tested in these analyses. While the emergence of telehealth replaced many of the in-person services in outpatient settings for people with IDD and IDD and SMI, we found that telehealth had less of an impact on in-person services for the IDD cohort, with inpatient, intensive outpatient and ED utilization staying relatively steady for this group, particularly for adults, over the four-year period.

### Limitations

Limitations of this study include the use of data from only four states, which are not representative of the U.S. In addition, we only accessed Medicaid members who did not have dual coverage by Medicare or other insurers, since we needed to have complete access to all health service utilization records. Our data source included people with one or more billed claims during the time periods; we included people with any type of billed claim to help broaden the study population, but the study is still at risk of excluding people who had no billed claims for any year, or whose Medicaid coverage terminated and reinitiated over the course of the study years. Finally, we did not have access to numerous potential confounders that could be associated with both the diagnostic groups being studied and the health services received. These potential confounders include individual characteristics (race, marital status, residential type, social supports, other coexisting conditions, and disability services) and characteristics of the medical service area in which the individuals lived (physician to population ratio, psychiatrist to population ratio, hospital type and size, and other psychiatric services). Most importantly, we did not have access to data about the quality of care received.

### Policy relevance

Due to the separation of state level developmental disability agencies from state mental health agencies (SMHAs) there has been a longstanding concern about the services accessible to children and adults with dual IDD and mental illness diagnoses, despite the evidence that people with IDD have higher rates of serious mental illness (9). Since the 1980s when home and community-based services (HCBS) waivers were introduced for Medicaid eligible persons with IDD, the mental illness supports afforded to this group were not clearly defined. Additionally, the support services of SMHAs do not typically address the needs of people with IDD.

The National Association for the Dually Diagnosed (NADD) focuses on persons with IDD and mental illness and provides practice guidelines in collaboration with the American Psychiatric Association (APA). The second edition of the Diagnostic Manual—Intellectual Disability (DM-ID-2) provides guidelines for treatment for the dually diagnosed, although having the needed infrastructure to provide both Emergency Department and Inpatient Hospital services to this population remains an ongoing challenge (13). The findings from this study of four states, with varying HCBS waiver offerings and differences in state Medicaid plans, provide evidence that people with IDD and SMI accessed hospital care for SMI at levels that exceeded those who had SMI only or IDD only. This finding is responsive to the call to action from the 2022 Special Article published in *Psychiatric Services* (13). However, although access to care for the dually diagnosed is being provided in these four states, both prior to and during the pandemic period, this study does not shed light on the quality of care. We acknowledge this study did not capture small area differences in clinical practice (13) and community characteristics that contribute to service utilization. Nonetheless, this study provides a foundation for tracking access to mental health care for children and adults up to age 45 years, with IDD and SMI who are insured by Medicaid. Our findings suggest mental health care is being provided equitably to Medicaid members with both IDD and SMI during the study period 2018–2021.

## Data Availability

The datasets presented in this article are not readily available because data is subject to the restrictions of each state Medicaid agency and thereby datasets cannot be shared publicly. Requests to access the datasets should be directed to Emily Lauer, emily.lauer@umassmed.edu.

## Author Disclaimer

The research and associated views presented in this paper are those of the authors and do not reflect the official policy of the Centers for Disease Control and Prevention, nor the official policy or position of any of the state Medicaid agencies or state Departments of Health. The views and opinions expressed in this article are those of the author(s) and do not necessarily reflect the official policy or position of the New York State Department of Health. Examples of analysis performed within this article are only examples. They should not be utilized in real-world analytic products.

## References

[1] CDC. Office of policy, performance, and evaluation. Health impact in 5 years (2024). Available online at: https://www.cdc.gov/policy/hi-5/index.html (Accessed July 22, 2025).

[2] Magaña S Parish S Morales MA Li H Fujiura GT. Racial and Ethnic Disparities among Adults with Intellectual and Developmental Disabilities. Silver Spring: Association of University Centers on Disabilities (2015).

[3] Hong C Hoskin J Berteau LK Schamel JT Wu ESC King AR. Violence victimization, homelessness, and severe mental illness among people who use opioids in three U.S. cities. J Interpers Violence. (2023) 38(19–20):11165–85. 10.1177/08862605231179720 37462229 PMC10466992

[4] Substance Abuse and Mental Health Services Administration. Key Substance Use and Mental Health Indicators in the United States: Results from the 2023 National Survey on Drug Use and Health. Center for Behavioral Health Statistics and Quality, Substance Abuse and Mental Health Services Administration (2024) (Rapport no HHS Publication No. PEP24-07-021, NSDUH Series H-59). Available online at: https://www.samhsa.gov/data/report/2023-nsduh-annual-national-report (Accessed January 7, 2025).

[5] Shaw KA. Prevalence and early identification of autism Spectrum disorder among children aged 4 and 8 years—autism and developmental disabilities monitoring network, 16 sites, United States, 2022. MMWR Surveill Summ. (2025) 74:1–22. 10.15585/mmwr.ss7402a1 40232988 PMC12011386

[6] Fletcher JM Miciak J. Assessment of specific learning disabilities and intellectual disabilities. Assessment. (2024) 31(1):53–74. 10.1177/10731911231194992 37671726 PMC10795803

[7] American Association on Intellectual and Developmental Disabilities. Intellectual Disability: Definition, Classification, and Systems of Supports, 11th ed. of AAIDD Definition Manual. Washington, DC, US: American Association on Intellectual and Developmental Disabilities (2010). p. xvi, 259.

[8] Whitney DG Peterson MD. US National and state-level prevalence of mental health disorders and disparities of mental health care use in children. JAMA Pediatr. (2019) 173(4):389–91. 10.1001/jamapediatrics.2018.5399 30742204 PMC6450272

[9] Buckley N Glasson EJ Chen W Epstein A Leonard H Skoss R. Prevalence estimates of mental health problems in children and adolescents with intellectual disability: a systematic review and meta-analysis. Aust N Z J Psychiatry. (2020) 54(10):970–84. 10.1177/0004867420924101 32475125

[10] Harris JC. Intellectual Disability: Understanding Its Development, Causes, Classification, Evaluation, and Treatment. USA: Oxford University Press (2006).

[11] Scott HM Havercamp SM. Mental health for people with intellectual disability: the impact of stress and social support. Am J Intellect Dev Disabil. (2014) 119(6):552–64. 10.1352/1944-7558-119.6.552 25354124

[12] Esler A Hewitt A Hall-Lande J Pettingell SL Houseworth J. Psychotropic medication use for adults with autism Spectrum disorder who receive services and supports through adult developmental disability services in the United States. J Autism Dev Disord. (2019) 49(6):2291–303. 10.1007/s10803-019-03903-7 30706350

[13] Fletcher R Barnhill J Cooper S-A. Diagnostic Manual—Intellectual Disability (DM-ID-2): A Textbook of Diagnosis of Mental Disorders in Persons with Intellectual Disability. 2nd ed. Kingston, New York: NADD (2017).

[14] Pinals DA Hovermale L Mauch D Anacker L. Persons with intellectual and developmental disabilities in the mental health system: part 1. Clinical considerations. Psychiatr Serv Wash DC. (2022) 73(3):313–20. 10.1176/appi.ps.201900504 34346730

[15] Pro G Giano Z Camplain R Haberstroh S Camplain C Wheeler D. The role of state medicaid expansions in integrating comprehensive mental health services into opioid treatment programs: differences across the rural/urban Continuum. Community Ment Health J. (2021) 57(6):1017–22. 10.1007/s10597-020-00719-z 33033972

[16] Buchanan RJ Schiffer R Wang S Stuifbergen A Chakravorty B Zhu L. Satisfaction with mental health care among people with multiple sclerosis in urban and rural areas. Psychiatr Serv Wash DC. (2006) 57(8):1206–9. 10.1176/ps.2006.57.8.1206 16870975

[17] Chapman DP Perry GS. The vital link between chronic disease and depressive disorders. Prev Chronic Dis. (2005) 2(1):1–10. Available online at: https://www.cdc.gov/pcd/issues/2005/jan/04_0066.htm PMC1323317 15670467

[18] Mental Health and Mental Disorders. Healthy People 2030 | odphp.health.gov. Available online at: https://odphp.health.gov/healthypeople/objectives-and-data/browse-objectives/mental-health-and-mental-disorders (Accessed July 22, 2025).

[19] Alliance for Connected Care. COVID-19 State Telehealth and Licensure Expansion Dashboard. Alliance for Connected Care (2020). (Accessed July 22, 2025).

[20] Mack B Whetsell H Graves J. Mental health in rural areas. National Rural Helath Association. (National Rural Helath Association Policy Brief) (2022).

[21] Andrilla CHA Patterson DG Garberson LA Coulthard C Larson EH. Geographic variation in the supply of selected behavioral health providers. Am J Prev Med. (2018) 54(6 Suppl 3):S199–207. 10.1016/j.amepre.2018.01.004 29779543

[22] McDermott S Royer J Cope T Lindgren S Momany E Lee JC. Using medicaid data to characterize persons with intellectual and developmental disabilities in five U.S. states. Am J Intellect Dev Disabil. (2018) 123(4):371–81. 10.1352/1944-7558-123.4.371 29949427

[23] Chronic Conditions Data Warehouse. Other chronic health, mental health, and potentially disabling conditions. Available online at: https://www2.ccwdata.org/condition-categories-other (Accessed July 22, 2025).

[24] AHRQ HCUP. Research tools. Available online at: https://hcup-us.ahrq.gov/tools_software.jsp (Accessed July 22, 2025).

[25] NCQA. HEDIS MY 2020 & MY 2021 Volume 2 (epub). Available online at: https://store.ncqa.org/hedis-my-2020-my-2021-volume-2-epub.html (Accessed July 22, 2025).

[26] SAS Institute Inc. SAS/STAT® 9.4. Cary, NC: SAS Institute Inc (2010).

[27] CMS. Ambulance fee schedule public use files. CMS. Available online at: https://www.cms.gov/medicare/payment/fee-schedules/ambulance/ambulance-fee-schedule-public-use-files (Accessed July 22, 2025).

[28] American Hospital Association. New/Modifications to the Place of Service (POS) Codes for Telehealth. American Hospital Association (2021).

[29] HCUP. Clinical classifications software refined (CCSR). Available online at: https://hcup-us.ahrq.gov/toolssoftware/ccsr/ccs_refined.jsp (Accessed July 22, 2025).

[30] Simmonds MC Higgins JP. A general framework for the use of logistic regression models in meta-analysis. Stat Methods Med Res. (2016) 25(6):2858–77. 10.1177/0962280214534409 24823642

[31] Harrier M Cuijpers P Furukawa T Ebert DD. Doing Meta-analysis in R. Boca Raton, FL and London: Chapman & Hall/CRC Press (2021). Available online at: https://bookdown.org/MathiasHarrer/Doing_Meta_Analysis_in_R/ (Accesed January 19, 2021).

[32] Veroniki AA Jackson D Viechtbauer W Bender R Bowden J Knapp G. Methods to estimate the between-study variance and its uncertainty in meta-analysis. Res Synth Methods. (2016) 7(1):55–79. 10.1002/jrsm.1164 26332144 PMC4950030

[33] Ahuja A Martin J Langley K Thapar A. Intellectual disability in children with attention deficit hyperactivity disorder. J Pediatr. (2013) 163(3):890–5.e1. 10.1016/j.jpeds.2013.02.043 23608559 PMC4078221

[34] Fleury M-J Ngui AN Bamvita J-M Grenier G Caron J. Predictors of healthcare service utilization for mental health reasons. Int J Environ Res Public Health. (2014) 11(10):10559–86. 10.3390/ijerph111010559 25321874 PMC4210995

[35] Green L McNeil K Korossy M Boyd K Grier E Ketchell M. HELP For behaviours that challenge in adults with intellectual and developmental disabilities. Can Fam Physician. (2018) 64(Suppl 2):S23–31. PMID: . 29650741 PMC5906788

[36] Charlot L Abend S Ravin P Mastis K Hunt A Deutsch C. Non-psychiatric health problems among psychiatric inpatients with intellectual disabilities. J Intellect Disabil Res. (2011) 55(2):199–209. 10.1111/j.1365-2788.2010.01294.x 20546095 PMC3646333

